# Effect of Collagen Peptide, Alone and in Combination with Calcium Citrate, on Bone Loss in Tail-Suspended Rats

**DOI:** 10.3390/molecules25040782

**Published:** 2020-02-12

**Authors:** Junli Liu, Jianing Wang, Yanchuan Guo

**Affiliations:** Key Laboratory of Photochemical Conversion and Optoelectronic Materials, Technical Institute of Physics and Chemistry, Chinese Academy of Science, Beijing 100190, China; junliliu306@163.com (J.L.); wangjianing@mail.ipc.ac.cn (J.W.)

**Keywords:** collagen peptide, calcium citrate, bone loss, tail-suspended rats

## Abstract

Oral administration of bovine collagen peptide (CP) combined with calcium citrate (CC) has been found to inhibit bone loss in ovariectomized rats. However, the protective effects of CP and CP–CC against bone loss have not been investigated in a tail-suspension simulated microgravity (SMG) rat model. Adult Sprague-Dawley rats (*n* = 40) were randomly divided into five groups (*n* = 8): a control group with normal gravity, a SMG control group, and three SMG groups that underwent once-daily gastric gavage with CP (750 mg/kg body weight), CC (75 mg/kg body weight) or CP–CC (750 and 75 mg/kg body weight, respectively) for 28 days. After sacrifice, the femurs were analyzed by dual-energy X-ray absorptiometry, three-point bending mechanical tests, microcomputed tomography, and serum bone metabolic markers. Neither CP nor CP–CC treatment significantly inhibited bone loss in SMG rats, as assessed by dual-energy X-ray absorptiometry and three-point bending mechanical tests. However, both CP and CP–CC treatment were associated with partial prevention of the hind limb unloading-induced deterioration of bone microarchitecture, as demonstrated by improvements in trabecular number and trabecular separation. CP–CC treatment increased serum osteocalcin levels. Dietary supplementation with CP or CP–CC may represent an adjunct strategy to reduce the risk of fracture in astronauts.

## 1. Introduction

The development and maintenance of bone structure depends on mechanical stimulation. Numerous studies have demonstrated that mechanical loading promotes bone formation, whereas the absence of mechanical stimulation decreases bone mass [1]. During space flight, astronauts experience microgravity that leads to serious physiological changes, one of the most prominent being bone loss, which increases fracture risk [2]. Long-term exposure to microgravity is associated with increased bone resorption and decreased bone formation, with a reduction in bone mineral density (BMD) of approximately 2% after one month, equivalent to annual bone loss in postmenopausal women [3].

Drug intervention is not routinely used in space flight and exercise has been combined with nutrition improvement. However, because of the lack of mechanical load or duration of space flight, osteoblast stimulation is insufficient to maintain bone mass [4]. This has led to a focus on pharmaceutical interventions such as osteoporosis drugs, but their potential to prevent bone loss in space remains to be clarified. In particular, the effects of different drugs, alone or combined, and dose–response relationships for improvements in bone quality and regeneration have not been investigated. Currently available therapeutic agents that are conventionally used for the prevention of bone loss have several side effects [5]. Therefore, novel therapeutic approaches are being explored, which have fewer side effects, while effectively minimizing the loss of bone mass.

Collagen, which is a major component of all tissues can be produced from various sources. For example, they can be extracted from sea animals such as shark, marine sponges [6,7]. In addition, they can be extracted from terrestrial animals such as porcine, bovine animals. However, collagen is mostly produced from pork skin and bovine bones. Moreover, bovine collagen is currently widely used for many applications such as foods and cosmetics [6].

Collagen peptide (CP), known as collagen hydrolysates, is mainly composed of mixtures of peptides obtained by partial hydrolysis of gelatins [8]. Due to its higher digestive absorption and safety, oral supplementation with CP for the restoration of bone joints has gained increasing scientific attention [9]. Daily doses of 150 or 500 mg/kg of CP for up to three months significantly prevented bone loss in ovariectomized (OVX) rats compared with control rats [10]. CP also improved vertebral composition and biomechanical strength, and increased the quantity and volume ratio of lumbar trabecular bone, which demonstrated its effect on bone protection [8]. In postmenopausal women with osteopenia, administration of calcium–collagen chelate supplements was found to improve bone mineral density and increase the rate of bone formation and bone resorption [11].

A previous study in OVX rats demonstrated that daily doses of either CP alone (750 mg/kg) or CP–calcium citrate (CC; 750–75 mg/kg) had an osteoprotective effect by inhibiting the loss of bone mineral density. Moreover, CP–CC suppressed trabecular bone loss and improved the microarchitecture of the distal femur [12]. However, the effect of CP on prevention and restoration of bone loss induced by microgravity has not been reported. The main purpose of this study was to observe the effects of oral administration of CP, alone and in combination with CC, on bone structure and bone metabolism in rats under hind limb unloading simulated microgravity (SMG), and to provide a theoretical basis for the use of CP–CC to prevent and treat microgravity-related osteoporosis.

## 2. Results

### 2.1. Effect of Tail Suspension on Body Weight

**Figure 1:**
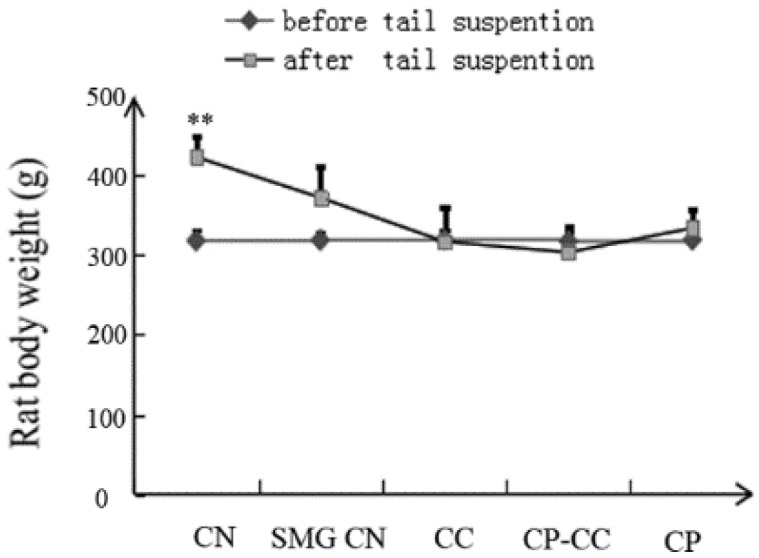
Body weight of rats before and after tail suspension (*n* = 8): CN, normal gravity control group; SMG CN, tail-suspended simulated microgravity control (SMG) groups; CC, SMG rats treated once-daily with 75 mg/kg calcium citrate (CC); CP–CC, SMG rats treated once-daily with 750 mg/kg bovine collagen peptide (CP) combined with 75 mg/kg CC; and CP, SMG rats treated once-daily with 750 mg/kg bovine CP. ** *p* < 0.01 vs. SMG CN.

Before tail suspension, there was no difference in the body weight of rats in the CN group and the four SMG groups (Figure 1). However, after 28 days of tail suspension, all SMG groups had lower body weight than the CN group (*p =* 0.000).

### 2.2. Bone Mineral Density Assessment

**Figure 2:**
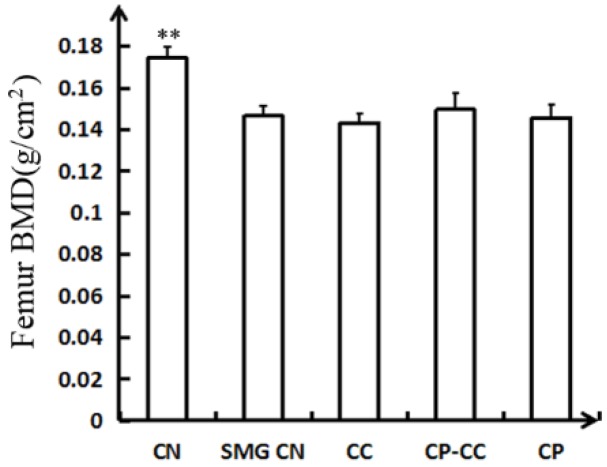
Femoral BMD determined by dual-energy X-ray absorptiometry analysis (*n* = 6) CN, normal gravity control group; SMG CN, tail-suspended simulated microgravity control (SMG) groups; CC, SMG rats treated once-daily with 75 mg/kg calcium citrate (CC); CP–CC, SMG rats treated once-daily with 750 mg/kg bovine collagen peptide (CP) combined with 75 mg/kg CC; and CP, SMG rats treated once-daily with 750 mg/kg bovine CP. ** *p* <0.01 vs. SMG CN.

Changes in femoral bone mineral density, as determined by dual-energy X-ray absorptiometry analysis, are shown in Figure 2. The SMG group had a 17.82% reduction in femoral bone mineral density compared with the CN group (*p =* 0.000). No significant improvement in the femoral bone mineral density of tail-suspended rats was observed after oral administration of CC, CP, or CP–CC.

### 2.3. Biomechanical Performance

Table 1 summarizes the maximum force to failure, deformation at hardness, and hardness work cycle values for the CN, SMG CN, SMG CC, SMG CP, and SMG CP–CC groups. Maximum force applied to failure in the SMG groups was 95.25 ± 18.64 N compared with 139.80 ± 24.73 N (*p* = 0.000) in the CN group, with an average decline of 31.65% in the SMG groups over the 28-day period. Maximum force applied to failure in the CC and CP–CC groups was 104.235 ± 17.868 and 99.442 ± 18.639 N, respectively, with a slight increase after tail suspension for 28 days. Deformation at hardness and femoral energy absorption were not significantly different among the four SMG groups.

### 2.4. CP–CC Treatment Inhibited Microgravity-Induced Deterioration of Bone Microarchitecture

**Figure 3:**
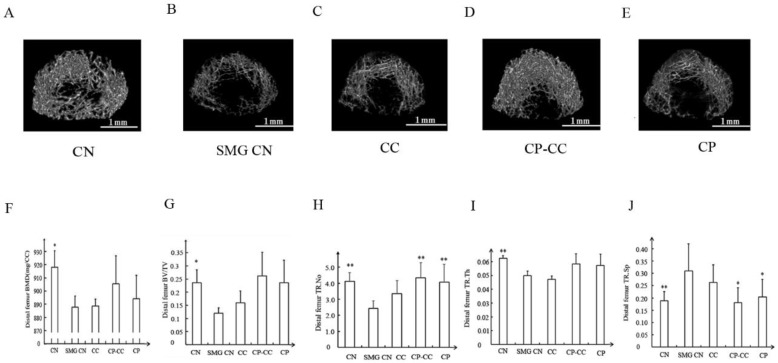
Representative three-dimensional microcomputed images (**A–E**) and (**F–J**, BMD, BV/TV, TR.N, TR.Th, TR.Sp) analysis of the microstructural indices of distal femurs in each group (*n* = 5) CN, normal gravity control group; SMG CN, tail-suspended simulated microgravity (SMG) control groups; CC, SMG rats treated once-daily with 75 mg/kg calcium citrate (CC); CP–CC, SMG rats treated once-daily with 750 mg/kg bovine collagen peptide (CP) combined with 75 mg/kg CC; CP, SMG rats treated once-daily with 750 mg/kg bovine CP. BV/TV, bone volume/tissue volume; Tb.N (TR.No), trabecular number; Tb.Th (TR.Th), trabecular thickness; Tb.Sp (TR.Sp), trabecular separation. * *p* < 0.05 vs. SMG group. ** *p* < 0.01 vs. SMG group. Scale bars, 1 mm.

Three-dimensional microcomputed tomography images of the distal femurs in all groups are shown in Figure 3. The CN group showed dense, intact trabecular bone, whereas the area and density of trabecular bone was reduced in the SMG CN group. Oral administration of CP or CP–CC improved bone microarchitecture and density, with thicker trabecular bone, indicating that bone loss was ameliorated.

Analysis of microstructural indices demonstrated that, compared with CN rats, the SMG CN rats had reductions in bone mineral density (3.29%, *p =* 0.029), BV/TV (91.48%, *p =* 0.026), Tb.N (41.12%, *p =* 0.006), and Tb.Th (20.63%, *p =* 0.005). Treatment with CP or CP–CC did not affect the reductions in bone mineral density, BV/TV, and Tb.Th compared with SMG CN rats; in contrast, Tb.N was enhanced by CP (67.52%, *p* = 0.001) and CP–CC (79.17%, *p* = 0.004). However, there were no differences between SMG CN and SMG CC rats. In the SMG rats, CP and CP–CC treatment decreased Tb.Sp by 34.52% (*p* = 0.05) and 41.94% (*p* = 0.02), respectively, compared with SMG CN rats, which had greater Tb.Sp than CN rats (38.7%, *p* = 0.021).

### 2.5. Serum Levels of Bone Turnover Biomarkers

Bone turnover was evaluated by serum levels of biomarkers of resorption (CTX and TRAP-5b) and formation (ALP, osteocalcin, and PINP). After four weeks, serum levels of Ca, ALP, and osteocalcin in the SMG groups were lower than in the CN group (*p* < 0.05, Table 2), except for osteocalcin levels in the SMG CP–CC group. After oral administration of CP–CC, serum osteocalcin levels of SMG rats were significantly higher than those of the SMG CN and other SMG treatment groups. PINP levels in SMG CN and SMG CP rats were significantly higher than in CN rats, whereas treatment with CC inhibited the increase in PINP (*p* < 0.05).

## 3. Discussion

Bone is a complex tissue. Hydroxyapatite salts (calcium and phosphorus) with collagen form a unique matrix that plays an important role in bone hardness. In addition, the Ca/P ratio in bones is vital for osteoporosis and may provide high reliability for diagnosis, prevention, and treatment of bone disorders. Collagen fibril diameter is related to bone site and Ca/P ratio. Ca/P ratio can serve as a reliable index of bone quality [13].

CP-based drugs play a role in the prevention and treatment of osteoporosis as orally administered, intestinally absorbed forms [14]. During space flight, weightlessness leads to calcium deficiency. High calcium intake from dietary supplementation does not affect bone metabolism, but prevents an elevation in serum calcium levels through increased calcitriol levels [15]. Therefore, intragastric administration was thought to be the most appropriate delivery route for bovine CP compounds combined with CC because of relative proximity to the pathologic process in the in vivo environment.

The results of this study show that rats in all SMG groups had significantly lower body weight than rats in the CN group, which is consistent with previous reports [16,17]. This may be related to loss of water electrolytes and loss of appetite caused by redistribution of body fluid under SMG.

Conventionally, the diagnosis and treatment of osteoporosis is assessed by bone mineral density, as measured by dual-energy X-ray absorptiometry [18]. Evaluation of bone biomechanical properties is indispensable to determine the quality of bone, and the intensity of bone fracture directly correlates to the relationship between the structure of the bone and the strength and hardness of the bone [19,20,21]. The present data show that SMG caused marked reductions in bone mineral density and femoral fracture strength of rats, which is consistent with previous reports [2,22]. These findings demonstrate that real or simulated weightlessness can cause bone changes, which are characterized by a decrease in cortical bone and cancellous bone formation [23].

Previous studies found that CP–CC led to substantial improvements in the matrix structure and quality of trabecular bone in the femurs of ovariectomized rats [12]. Dual-energy X-ray absorptiometry and biomechanical tests showed that CP–CC had a significant effect on femoral bone mineral density and fracture strength of ovariectomized rats [24]. Therefore, in the present study, the effects of CP–CC on bone remodeling were evaluated in a rat model of SMG. However, neither CP alone nor in combination with CC inhibited bone loss in SMG rats, based on dual-energy X-ray absorptiometry and three-point bending mechanical test analyses. Furthermore, assessment of the femoral microarchitecture using microcomputed tomography revealed that neither CP nor CP–CC had obvious effects on bone mineral density, BV/TV, or Tb.Th in tail-suspended SMG rats. A possible reason may be that CP and CP–CC act primarily via stimulation of bone formation to inhibit bone loss [8]. However, microgravity causes uncoupling of formation and resorption in bone remodeling, which may contribute to bone loss [25,26].

Nevertheless, CP and CP–CC treatment partially ameliorated microgravity-induced deterioration of bone microarchitecture, as indicated by suppressing both the reduction in Tb.N and the increase in Tb.sp induced by hind limb unloading simulated microgravity. This result is consistent with a previous report that oral administration of CP or CP–CC inhibited bone loss in ovariectomized rats [12]. This may represent an adjunctive dietary strategy to reduce the risk of fracture in astronauts.

Some previous reports suggest that serum calcium levels are not altered by tail suspension [27,28]. On the contrary, calcium levels in the tail-suspended rats were significantly reduced in another previous study [22]. This implies that intestinal calcium absorption was reduced during tail suspension [29]. Serum concentrations of bone turnover markers are reflective of bone remodeling activity, and can potentially be used as surrogate markers of the rate of bone formation or bone resorption [30]. The present study also showed that ALP activity and osteocalcin levels were decreased under SMG conditions, which is consistent with a previous report [31,32], indicating that osteoblast activity was inhibited by microgravity [33]. The main function of osteocalcin is to maintain the normal mineralization rate of bone. Interesting, osteocalcin levels in CP–CC-treated SMG rats were similar to those of CN rats, demonstrating that CP–CC promotes osteocalcin levels of osteoblasts by hind limb unloading simulated microgravity. PINP is a well-established marker of bone formation, which is produced by formation of type I collagen, a major component of the bone matrix, by amino-terminal and carboxy-terminal splicing of type I procollagen in osteoblasts [34]. Conversely, β-CTX is a marker of bone resorption, reflecting the degradation of type I collagen by osteoclasts to produce amino-terminal and carboxy-terminal fragments [35]. After ovariectomy or orchidectomy, serum TRACP-5b levels, which reflect the number of osteoclasts rather than their activity [36], are expected to decline, and the histomorphometrically determined total number of osteoclasts in bone tissue is decreased owing to substantial bone loss [37]. Changes in serum PINP or β-CTX levels induced by microgravity have reported before [38]. High serum PINP levels in SMG or CP-treated rats in our study demonstrated that hind limb unloading promoted formation of type I collagen. However, treatment with CC significantly suppressed the hind limb unloading-induced increase in type I collagen formation. A possible reason for the lack of effects of CP–CC on serum PINP levels was that the actions of CP and CC counteracted one another. In this study, serum β-CTX levels were slightly reduced in tail-suspended rats, which is contrary to a previous report that CP–CC or CP supplementation inhibited the degradation of collagen in ovariectomized rats [22], and serum TRACP-5b levels of tail-suspended rats were not altered. It may be speculated that this is related to the time of blood sampling or the high extent of bone loss under hind limb unloading simulated microgravity.

This study has some limitations. CP–CC treatment did not display a dose–response effect, and the duration of tail suspension was relatively short. As a dietary supplement, the effective time of CP–CC treatment in ovariectomized rats is three months, while, in this study, tail suspension was only maintained for 28 days. Thus, differing results among studies may be due to the mechanisms underlying the two animal models, and, in the tail suspension model, rapid bone loss in the process of CP–CC is not caused by the changes of bone mineral density obviously. Second, continuous blood sampling was not performed for observation of bone turnover markers.

## 4. Materials and Methods

### 4.1. Animals and Treatment

Bovine bone CP (prepared by our laboratory) and CC (Dongtai Food Ingredients, Lianyungang, China) were used. Male, three-month-old Sprague-Dawley rats (*n* = 40, body weight: 300 ± 20 g) were obtained from the animal facility of the China Astronaut Research and Training Center (Beijing). Animals were maintained in cages under standard conditions (12-h light/dark cycle with free access to food and water). After feeding adaptation for seven days, the rats were divided into five groups (*n* = 8 each): a control group with normal gravity (CN), an untreated hind limb unloading simulated microgravity group (SMG CN), and three SMG groups that underwent once-daily treatment by gastric gavage with CC (75 mg/kg), CP (750 mg/kg), or CP–CC (750 and 75 mg/kg). Bovine CP and CC were dissolved in distilled water. The tail-suspended rats were fixed by the tail at a 30° head-down angle to mimic microgravity [39]. Briefly, the rats were individually caged, suspended by the tail using a strip of adhesive surgical tape attached to a chain hanging from a pulley, and subjected to hind limb unloading by tail suspension for 28 days. After sacrifice, serum was collected, and the bilateral femurs and tibiae were dissected and processed for dual-energy X-ray absorptiometry, three-point bending mechanical tests, microcomputed tomography, and evaluation of serum bone metabolic markers. All experimental procedures were approved by the Committees of Animal Ethics and Experimental Safety of the China Astronaut Research and Training Center.

### 4.2. Bone Mineral Density Assessment

The bone mineral density of femurs was measured by dual-energy X-ray absorptiometry equipped with appropriate software for small laboratory animals (GE Lunar PIXImus, GE Healthcare, Madison, WI, USA). All right femurs were placed in the same direction. Values were expressed as the observed mean (g/cm^2^) ± standard deviation (SD) of the whole group.

### 4.3. Biomechanical Testing

Biomechanical analysis was performed by three-point bending mechanical tests. Experiments were conducted using TexturePro CT V1.3 Build 14 (Brookfield Engineering Labs, Inc., Stoughton, MA, USA). Femora were placed horizontally on the frame on rounded edges at a distance of 20 mm. To minimize variability, the specimens were placed in a consistent position and orientation. Maximum force applied to failure, deformation at hardness and hardness work cycle values were recorded.

### 4.4. Microcomputed Tomography Analysis of the Distal Femur

The secondary spongiosa extracted from the left distal femurs of rats was scanned with a desktop, microcomputed tomography scanner (μCT40; Scanco Medical, Bruttisellen, Switzerland) using a voxel size of 10 μm, an X-ray tube voltage of 70 kVp, a current intensity of 114 μA, and an integration time of 600 ms. Briefly, slices were scanned at the region of the distal femur beginning at 0.1 mm from the most proximal aspect of the growth plate and extending proximally along the femur diaphysis. A volume of interest was manually drawn on each specimen. Microstructural measures included trabecular bone mineral density, bone volume per total volume (BV/TV), trabecular thickness (Tb.Th), trabecular number (Tb.N), and trabecular separation (Tb.Sp). Computation of these structural measures was performed using a previously described method [12].

### 4.5. Biochemical Serum Analysis

Blood was collected and serum were separated by centrifugation to determine alkaline phosphatase activity (ALP) using an autoanalyzer. Serum bone osteocalcin/bone GLA protein (BGP) content was measured with a carboxyglutamic acid radioimmunometric assay kit (BGP Radioimmunometric Assay, Beijing North Institute of Biological Technology, Beijing, China), according to the manufacturer’s protocol. Serum N-terminal propeptide of type I procollagen (PINP) was measured with a specific rat PINP enzyme immunoassay (Rat PINP EIA; IDS Ltd., UK). The Beta isomer of serum C-telopeptide of type I collagen (CTX) was measured by an ELISA specific for rat CTX (RatLaps ELISA; IDS. Serum tartrate-resistant acid phosphatase form 5b (TRACP-5b) was measured by an ELISA specific for rat TRACP-5b (RatTRAP Assay; IDS).

### 4.6. Statistical Analysis

All numerical data are expressed as means ± SD. Statistical analyses were performed using SPSS for Windows version 17.0 (IBM, Armonk, NY, USA). With sample size of 8 (*n* = 5 or 6), nonparametric statistical analysis were performed. After one-way ANOVA, least-significant differences or Dunnett’s post-hoc test was used to determine significant differences between groups. Values of *p* < 0.05 were considered to indicate statistical significance.

## 5. Conclusions

Bovine CP, alone or in combination with CC, did not inhibit bone loss in a tail-suspended rat model of microgravity. However, CP or CP–CC treatment partially ameliorated the microgravity-induced deterioration of bone microarchitecture. This may represent an adjunct dietary strategy to reduce the risk of fracture in astronauts and highly sedentary populations.

## Figures and Tables

**Table 1:** Femoral biomechanics analysis.

	CN	SMG CN	CC	CP–CC	CP
FFS	139.80 ± 24.73 **	95.25 ±1 8.64	104.24 ± 17.87	99.44 ± 16.19	97.10 ± 15.01
FD	7.39 ± 0.56	7.40 ± 0.68	7.48 ± 0.57	7.37 ± 0.58	7.4 ± 0.55
FEA	96.77 ± 42.94	67.478 ± 17.23	93.96 ± 24.37	76.94 ± 20.76	82.34 ± 10.85

FFS, Femoral fracture strength (N) FD, Femoral deformation (mm) FEA, Femoral energy absorption (mJ) CN, normal gravity control group; SMG CN, tail-suspended simulated microgravity (SMG) control groups; CC, SMG rats treated once-daily with 75 mg/kg calcium citrate (CC); CP–CC, SMG rats treated once-daily with 750 mg/kg bovine collagen peptide (CP) combined with 75 mg/kg CC; CP, SMG rats treated once-daily with 750 mg/kg bovine CP. Data are expressed as means ± SD (*n* = 6). ** *p* < 0.01 vs. SMG CN.

**Table 2:** Serum levels of bone turnover biomarkers analysis.

	CN	SMG CN	CC	CP–CC	CP
Ca	2.60 ± 0.11011	2.38 ± 0.14 ^##^	2.37 ± 0.08 ^##^	2.38 ± 0.08 ^##^	2.38 ± 0.05 ^##^
P	2.64 ± 0.22	2.66 ± 0.19	3.01 ± 0.41	2.86 ± 0.22	2.65 ± 0.41
ALP (U/L)	202.33 ± 21.05 **	155.86 ± 25.53 ^##^	174.57 ± 38.20 ^#^	148.33 ± 0.38 ^##^	162.00 ± 24.36 ^##^
Osteocalcin (ng/mL)	3.90 ± 0.82 **	2.66 ± 0.37 ^##^	2.70 ± 0.51 ^##^	3.96 ± 0.51 **	2.98 ± 0.70 ^##^
PINP (ng/mL)	34.00 ± 2.19 **	77.00 ± 5.29 ^##^	37.20 ± 2.17 **	51.20 ± 17.46	68.00 ± 8.81 ^##^
CTX (ng/mL)	66.14 ± 8.80	60.43 ± 12.00	64.71 ± 8.54	60.25 ± 9.30	65.00 ± 6.00
TRAP-5b	2.07 ± 0.45	2.18 ± 0.22	3.71 ± 1.88	4.29 ± 1.50	3.34 ± 1.28

CN, normal gravity control groups; SMG CN, tail-suspension simulated microgravity control groups; CC, SMG rats were administrated 75 mg/kg calcium citrate daily; CP–CC, SMG rats were administrated 750 mg/kg bovine collagen peptide combined with 75 mg/kg calcium citrate daily; CP, SMG rats were administrated 750 mg/kg bovine collagen peptide daily, as indicated. ALP, bone-specific alkaline phosphatase; PINP, procollagen type I N-terminal propeptide; CTX, carboxyterminal telopeptide of collagen type 1. Data are expressed as means ± SD (*n* = 8). ^#^ *p* < 0.05 vs. CN. ^##^ *p* < 0.01 vs. CN. ** *p* < 0.01 vs. SMG CN.
